# Emerging Biofabrication Techniques: A Review on Natural Polymers for Biomedical Applications

**DOI:** 10.3390/polym13081209

**Published:** 2021-04-08

**Authors:** María Puertas-Bartolomé, Ana Mora-Boza, Luis García-Fernández

**Affiliations:** 1 INM—Leibniz Institute for New Materials, Campus D2 2, 66123 Saarbrücken, Germany; 2 Saarland University, 66123 Saarbrücken, Germany; 3 Woodruff School of Mechanical Engineering and Petit Institute for Bioengineering and Bioscience, Georgia Institute of Technology, 315 Ferst Drive, 2310 IBB Building, Atlanta, GA 30332-0363, USA; 4 Institute of Polymer Science and Technology (ICTP-CSIC), Juan de la Cierva 3, 28006 Madrid, Spain; 5 Networking Biomedical Research Centre in Bioengineering, Biomaterials and Nanomedicine (CIBER-BBN), Monforte de Lemos 3-5, Pabellón 11, 28029 Madrid, Spain

**Keywords:** biofabrication, microfluidics, electrospinning, 3D printing, electrospraying, natural polymers, cell encapsulation

## Abstract

Natural polymers have been widely used for biomedical applications in recent decades. They offer the advantages of resembling the extracellular matrix of native tissues and retaining biochemical cues and properties necessary to enhance their biocompatibility, so they usually improve the cellular attachment and behavior and avoid immunological reactions. Moreover, they offer a rapid degradability through natural enzymatic or chemical processes. However, natural polymers present poor mechanical strength, which frequently makes the manipulation processes difficult. Recent advances in biofabrication, 3D printing, microfluidics, and cell-electrospinning allow the manufacturing of complex natural polymer matrixes with biophysical and structural properties similar to those of the extracellular matrix. In addition, these techniques offer the possibility of incorporating different cell lines into the fabrication process, a revolutionary strategy broadly explored in recent years to produce cell-laden scaffolds that can better mimic the properties of functional tissues. In this review, the use of 3D printing, microfluidics, and electrospinning approaches has been extensively investigated for the biofabrication of naturally derived polymer scaffolds with encapsulated cells intended for biomedical applications (e.g., cell therapies, bone and dental grafts, cardiovascular or musculoskeletal tissue regeneration, and wound healing).

## 1. Introduction 

Polymeric biomaterials have been developed to provide an artificial matrix that can mimic the cell microenvironment. This artificial matrix needs to provide appropriate biophysical and structural properties (e.g., stiffness, roughness, topography, and alignment) as well as biochemical cues (e.g., signaling, growth factors, and proteins) in order to promote the native capacity of cells to adhere, migrate, proliferate, and differentiate towards the growth of new tissue [1]. 

Natural polymers extracted from biological systems such as plants, microorganisms, algae, or animals have been used for decades in the biomedical field. These materials retain the biochemical cues and properties necessary to improve their biocompatibility and present similar structures to the extracellular matrix (ECM) of native tissues [2,3,4,5]. Therefore, they usually present good cellular attachment, improve cellular behavior, and avoid immunological reactions, although in some cases, these properties are limited due to batch variability within production and purification processes. The most common natural polymers used in biomedical applications include polysaccharides (e.g., alginate [5,6,7], hyaluronic acid [3,8], and chitosan [9,10]), proteins (e.g., collagen [11], silk [12,13], gelatin [14,15,16], and fibrin [17]), and bacterial polyesters (e.g., bacterial cellulose [18]). However, the poor mechanical strength of natural polymers frequently makes the manipulation and biofabrication process difficult. For this reason, the use of derivatives or blends with different polymers are usually required to obtain appropriate mechanical properties for their use. An example is the modification of gelatin with methacrylamide to obtain a photopolymerizable biomaterial that can be used for 3D bioprinting and microfluidics [19,20,21,22].

Actual biomedical challenges require the use of complex polymer matrixes that can mimic the native ECM and regenerate the lost or damaged tissues [23,24,25]. Recent advances in biofabrication techniques allow the production of a polymer matrix with biophysical and structural properties similar to the ECM, and its combination with different cell lines is capable of proliferating and differentiating into the desired tissue. Moreover, the incorporation of different growth factors or other biomolecules can improve the migration, growth, and differentiation of the cells [3,26].

Currently, numerous research lines for polymer matrix biofabrication follow two different strategies for the incorporation of the cells: (i) cell implantation on the previously formed polymer matrix and (ii) fabrication of a polymer matrix with encapsulated cells. 

The first strategy was used in the last decade, and it is restricted to the method of cell implantation. Normally, these systems do not present a good integration between cells and the polymer matrix, and their efficacy for tissue regeneration depends on the physical properties of the polymer matrix such as hydrophobicity, degradation rate, or stiffness [14,27]. Among the most used techniques, we can highlight layer-by-layer [28,29], melt molding [30], photolithography [31], and self-assembling [32].

The second strategy is the most investigated in recent years, since it allows the fabrication of advanced cell-laden structures with complex cellular microenvironments. Recently, some advanced techniques (i.e., microfluidics [33,34], electrospinning [10,35], and 3D printing [36,37]) allow the integration of cells directly into the polymer matrix with the adequate physical and biological properties to imitate the ECM of the desired tissue.

This review focuses on the biofabrication techniques of microfluidics, electrospinning, and 3D printing using natural polymers. These techniques have been recently explored to create polymer matrixes with embedded cells for biomedical applications, and they are in continuous evolution, as we are going to illustrate in the present review.

## 2. Microfluidics

**Figure 1:**
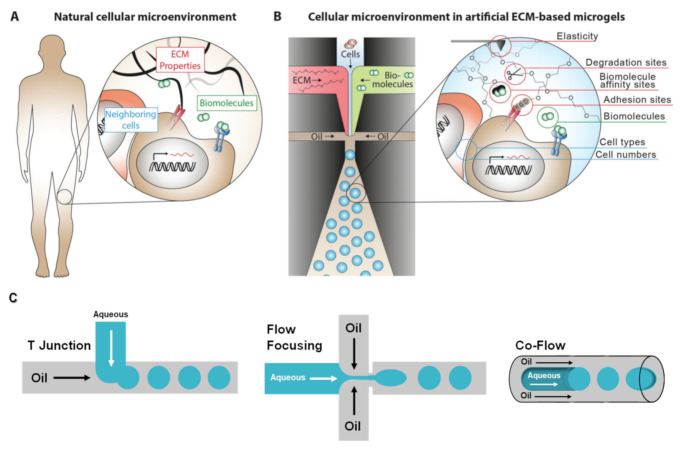
Recapitulating the natural cellular microenvironment in biomimetic microgels using droplet-based microfluidics. (**A**) The natural cellular microenvironment is composed of different cell types, ECM, and biomolecules such as growth factors. (**B**) Droplet-based microfluidics allows for versatile and high throughput generation of cell-laden microgels that can mimic the natural cellular environment. By mixing defined amounts of selected cells, ECM, and biomolecules, the microenvironment can be designed in a bottom-up approach with defined properties [34]. (**C**) Schematic illustration of different types of droplet generators, including T-Junction, flow-focusing, and co-flow (capillary) configurations. Adapted with permission from John Wiley and Sons Copyright^®^.

Microfluidics has emerged as a powerful tool for the high throughput generation of monodisperse microgels [33,34]. Microgels are defined as 3D-crosslinked particles that provide a porous polymeric network and can recapitulate the cellular microenvironment (i.e., ECM), mimicking in vivo conditions and diffusion of nutrients and metabolic waste [38,39,40] (Figure 1A,B). Specifically, microgels are fabricated in microfluidic devices by the generation of polymer droplets (i.e., droplet-based microfluidics) through water/oil emulsions followed by physical or chemical crosslinking. The most frequently used geometry configurations to generate the droplets in the devices are T-junction, flow-focusing, and co-flowing (or capillary) laminar streams, which are illustrated in Figure 1C [41,42,43,44]. Microgels are especially attractive as cell carriers, because their large surface-to-volume ratio promotes efficient mass transport and enhances cell-matrix interactions, but it is important to notice that cell microencapsulation requires a polymer network that ensures cell viability during microgel preparation and adequate crosslinking chemistry to form a polymer network [45]. Microfluidics technology provides a tight control over microgel chemical properties and composition by easily tuning the flow rates and components in the microfluidic channels, being a versatile biofabrication platform, where different crosslinking strategies can be applied [41]. As mentioned, the microdroplets generated in the microfluidic devices should undergo physical (e.g., electrostatic interaction, thermal gelation, and hydrogen bond interaction) or chemical crosslinking (photopolymerization, Michael addition, and enzymatic reaction) to form solidified microgels [38]. Physical or chemical gelation will be chosen based on different factors like the type of polymer, the strategy for tissue encapsulation, as well as the final biomedical application. In addition, different crosslinking mechanisms can be combined to fulfil the desired features of the microgel systems [38,43,46,47], and these crosslinking processes can take place inside the microfluidic device (in situ crosslinking) or after microgel collecting [38]. In this review, most recent examples of interesting processes for the microfluidics generation of cell-laden microgels prepared using natural polymers and different crosslinking strategies are exposed (Table 1).

### 2.1. Naturally Derived Polymer Used for the Preparation of Cell-Laden Microgels Using Microfluidics

#### 2.1.1. Alginate

Alginate is a classic polymer used for the generation of microgels through microfluidics [38]. Typically, an aqueous alginate solution is emulsified in an oil phase and crosslinked ionically with bivalent ions such as Ca^2+^, which can be found for example in CaCl_2_ or CaCO_3_. The ionic crosslinking process occurs immediately upon contact of alginate chains and Ca^2+^ ions [48]. Kumacheva and co-workers have reported the most representative works of alginate-based cell-laden microgels for the last years [43,46,57,58]. Alginate microgels can be prepared through an internal or external gelation approach [38]. In the internal crosslinking, also called in situ crosslinking methodology, alginate is exposed directly to the crosslinking agent, triggering gelation [59]. In the external approach, alginate droplets are firstly formed and then put into contact with the crosslinker solution [60]. This last approach provides a better control over the final morphology of the microgels [38].

Utech et al. [48] reported an interesting method for the fabrication of alginate microgels using a water-soluble calcium-ethylenediaminetetraacetic acid (calcium-EDTA) complex as a crosslinking agent. They were able to encapsulate individual MSCs with high cell viability due to the mild polymerization approach. Moreover, encapsulated MSCs grew and proliferated over two weeks. Encapsulation of MSCs in polymeric microgels is an excellent approach to improving cell persistence and immunomodulation [61]. Cell therapies based on MSCs are particularly interesting in ameliorating immune-related diseases and dysregulations, but they are limited due to short in vivo persistence [44,45,61,62]. Mao et al. [61] reported the encapsulation of MSCs in alginate-polylysine microgels using a microfluidic device. The encapsulated MSCs in their microgel formulation significantly increased their in vivo persistence after intravenous injection and responded to inflammatory cytokines, improving immunomodulatory effect of MSCs in a model of allogeneic transplantation.

Alginate has been also combined with synthetic polymers to fabricate microgels [33,63,64]. Chen et al. [33] synthetized a functional diblock copolymer, alginate-conjugated poly(N-isopropylacrylamide) (PNiPAM), to fabricate microgels in a flow-focusing device also using calcium-EDTA complex as a crosslinking agent. The permeability of the as obtained microgels could be modified by controlling temperature at low critical solution temperature (LCST), and the encapsulated human hepatocellular carcinoma cell (HepG2) showed high cell viability thanks to the mild conditions of the crosslinking process.

In recent years, other interesting microfluidic methodologies have been developed to overcome some limitations of conventional droplet-based microfluidics. This is the case for the work carried out by Cheng et al. [50], where an efficient centrifugal microfluidic system for controllable fabrication of simple structured alginate hydrogel beads and fibers was exposed. Among the advantages of centrifugal microfluidics, it is highlighted by the use of simple experiment facilities (i.e., a centrifuge) and the absence of an oil continuous phase and subsequent necessary washing steps. HepG2 cells were encapsulated in the developed alginate capsules and fibers, demonstrating the high validity of the method, and showing excellent potential for biomedical applications. Other studies have optimized a water-in-oil-in-water (W/O/W) double emulsion methodology to encapsulate cells. Chan et al. [11,35] developed a double emulsion platform to encapsulate rat hepatocytes and endothelial cells using a combination of alginate and collagen. The developed microgels provided an excellent physical support for the spheroids.

#### 2.1.2. Hyaluronic Acid

Hyaluronic acid-based building blocks have been prepared by pseudo Michael addition crosslinking in a microfluidic device [8,38]. Sideris et al. [8] reported a microfluidics methodology for the fabrication of hyaluronic acid microgels that could self-assemble to form a biodegradable scaffold through two orthogonal chemistries. Human dermal fibroblasts were seeded after microgel preparation, demonstrating good cell spreading after two days of culture. Ma et al. [51] developed a new hyaluronic acid derivative, furylamine and tyramine hyaluronic acid, that can be crosslinked using enzymatic crosslinking, Diels-Alder click chemistry, or a combination of both methods. The versatility of this strategy provided control over crosslinking time and elasticity by simply switching the crosslinking strategy. After evaluating the mechanical properties, gelation time, microgel size, swelling, enzymatic degradation, and bioactivity of the obtained microgels, the group concluded that the microgels synthetized through the combination of both crosslinking methods were the most promising candidates for cell encapsulation and delivery, since the use of the strategies alone resulted in low elasticity and poor cell encapsulation performance.

#### 2.1.3. Chitosan

Jang et al. [52] proposed a new in situ crosslinking methodology for the fabrication of microgels by merging two droplets of different viscosities in an asymmetric cross-junction microfluidic device. Thus, oxidized dextran (ODX) and N-carboxymethyl chitosan (N-CEC) were mixed to undergo an in situ crosslinking via a Schiff base reaction, resulting in microgel formation. This asymmetric cross-junction geometry was an interesting approach to overcoming the high surface tension of microdroplets of contrasting viscosities, which usually requires significant surfactant concentrations. In addition, the crosslinking methodology allowed the encapsulation of NIH-3T3 fibroblasts, which showed high viability after two days of culture, demonstrating the biocompatibility of the entire process. Mora-Boza et al. [45] reported the fabrication of hMSCs-laden microgels, applying also an in situ crosslinking approach for chitosan lactate (ChLA), a water-soluble chitosan derivative. The ionotropic gelation was based on a combination of glycerylphytate (G_1_Phy) and tripolyphosphate (TPP) as ionic crosslinkers, obtaining polymeric microgels with homogeneous size distribution between 104 and 127 µm. The authors demonstrated that the presence of G_1_Phy, which has been recognized as a potent antioxidant and bioactive compound [65], supported encapsulated hMSC viability over time and modulated hMSCs secretome at adverse conditions, resulting in an appealing cell delivery platform for hMSCs therapy applications.

#### 2.1.4. Gelatin

Photocrosslinkable gelatin derivative, methacrylated gelatin (GelMA), has been widely applied for the preparation of cell-laden microgels through microfluidics [15,38]. Lee et al. [20] developed microtissues containing macrophages through flow-focusing microfluidics using GelMA as a macromer solution. The macrophages’ viability was well maintained, and mechanical properties of the microgels could be controlled through GelMA concentration, which had a strong influence on the proliferation and polarization of the encapsulated cells. A similar methodology was applied by Weitz’s group to encapsulate bone marrow derived MSCs. The authors demonstrated that the encapsulated cells migrated to the surface of the microgels after four weeks of culture, indicating their capacity to participate in regenerative processes. Moreover, they demonstrated in vivo osteogenic potential by increasing the percentage of calcium deposits and expression of bone-related proteins like BMP-2 [53].

Photocrosslinkable gelatin has also been applied in combination with other synthetic polymers like polyethyleneglycol (PEG) [21,22]. Seeto et al. [21] used a custom designed microfluidic device with a T-junction geometry that allowed the production of microgels with a wide range of diameters from 300 to 1100 µm. The group used a combination of poly(ethylene glycol) diacrylate (PEGDA), poly(ethylene glycol)-fibrinogen (PF), and GelMA, which underwent fast photocrosslinking using a full spectrum light source and Eosin Y as a light photoinitiator. High cellular densities of different cell lines, including horse endothelial colony forming cells (ECFCs), breast cancer cells, or human induced pluripotent stem cells (hiPSCs), were encapsulated in the microspheres, showing good cell distribution, high viability, and functional cellular activities. Forsythe’s group combined PEG with another photocrosslinkable derivative of gelatin, gelatin norbornene (GelNB), to fabricate cell-laden microgels using visible light. The encapsulated hBMSCs in the GelNB-PEG microspheres demonstrated chondrogenesis properties when incubated with chondroinductive media, including significant upregulation of collagen-II expression in comparison to bulk hydrogels [22]. Cartilage repair properties have also been observed in the stem cell-laden microgels reported by Feng et al. [16]. In their work, thiolated gelatin and vinyl sulfonated hyaluronic acid were mixed in a microfluidic device to generate microgels through a thiol-Michael addition reaction. Encapsulated bMSCs showed excellent viability, proliferation, and chondrogenic properties. Furthermore, the in vivo experiments demonstrated that the cell-laden microgels were injectable and could self-assemble into cartilage-like structures, providing an effective method for cartilage tissue regeneration, since they were able to inhibit vascularization and hypertrophy.

#### 2.1.5. Dextran

Dextran application in microfluidics technology has also been explored [38,54,55]. Henke et al. [54] developed very stable dextran-tyramine microgels through enzymatic crosslinking for hMSCs encapsulation, which demonstrated significantly higher cell viability in comparison to PEGDA and alginate microgels. Dextran-based microgels supported cells’ metabolic activity and allowed cell analysis for 28 days of culture. Liu et al. [55] combined dextran with PEG to generate water-in-water droplets in a cross-flow microfluidic device. This strategy allowed avoiding the use of organic solvent and its subsequent removal. The droplets could be also used as templates for the fabrication of alginate microgels. Moreover, the platform was demonstrated to be a promising system for tissue engineering applications, since the encapsulated rat pancreatic islets maintained high viability and the function of insulin secretion after seven days of culture.

#### 2.1.6. Heparin

Heparin has been combined with PEG to generate bioactive microgels via Michael addition to encapsulate and enhance the differentiation of mESCs [38,56]. Siltanen et al. [56] mixed heparin methacrylate and PEG diacrylate monomers with 8-arm PEG-thiol to fabricate bioactive microgels that provided a suitable environment for endodermal differentiation. The authors also incorporated growth factors FGF-2 and Nodal to evaluate the differentiation processes of the encapsulated cells, showing that 3D differentiation processes significantly upregulated the expression levels of endoderm markers.

### 2.2. Future Perspectives in Fabrication of Cell-Laden Microgels through Microfluidics

Microfluidics is a versatile technology to generate monodisperse cell-laden microgels, whose properties can be easily tuned if a sensible selection of biomaterials and crosslinking strategies is applied [38,47,66]. These microgels can be applied as building blocks that can self-assemble into mesoscale tissue structures and replicate structures of native tissues [8,67,68]. Nevertheless, some limitations must be overcome before clinical implementation of microfluidic microgels see a bright future [38]. One of the foremost concerns is related to the scalability of current microfluidic strategies. A higher and more robust mass production of cell-laden microgels is necessary to scale-up this technology and be able to obtain macroscale tissue assemblies that can be implemented in the clinic [38,67]. Therefore, new devices that can support large-scale production of microgels with complex geometries, such as core-shell morphology, are needed [38]. Another limitation of current cell-laden microgels is the lack of proper vascularization. Vascularization is essential for effective tissue implantation. Thus, a biomimetic tissue construct should contain essential elements like different cell lines, ECM components, and a vasculature network to maintain cellular interaction and normal tissue function [38,69,70,71]. Regarding this issue, many efforts are being made in recent years to develop devices and strategies to fabricate vasculature in the hydrogels or incorporate well-perfused vasculature networks through microfluidics systems [38].

## 3. Cell-Electrospinning (CE) and Bio-Electrospraying (BES)

Electrospinning is a well-known technology that allows the fabrication of micro/nanofiber scaffolds using different synthetic and natural polymers [72,73,74,75]. A typical electrospinning set up requires a nozzle tip, a high voltage supply, a pump to control flow rate, and a grounded collector. The process is based on the application of an electric field between the metallic syringe needle and the grounded collector, while the polymer solution is pumped out from the needle at a controlled rate. A conical shape called a “Taylor cone” is generated at the end of the nozzle. When the electrostatic forces within the cone are higher than the surface tension of the solution, the polymer generates a jet, and it is accelerated toward the collector plate, forming a randomly oriented nanofibers mat [75,76].

The concept of electrospinning was first introduced by Anton Formhals in the 1930s. Since then, it has been widely applied in numerous fields such as textiles, agriculture, filtration, sensors, and the biomedical area [24,77,78,79,80,81,82,83]. Specially, this technique has had a great impact on the area of tissue engineering, since it presents several advantages: the nanofiber mats can create complex structures that can simulate the native structure of the ECM, promoting the normal functions of cells; it has an easy manufacture and availability; the high porosity and high surface area due to the nano size of the fibers enhance cellular activities such as cell attachment proliferation and differentiation [84,85,86,87,88,89].

**Figure 2:**
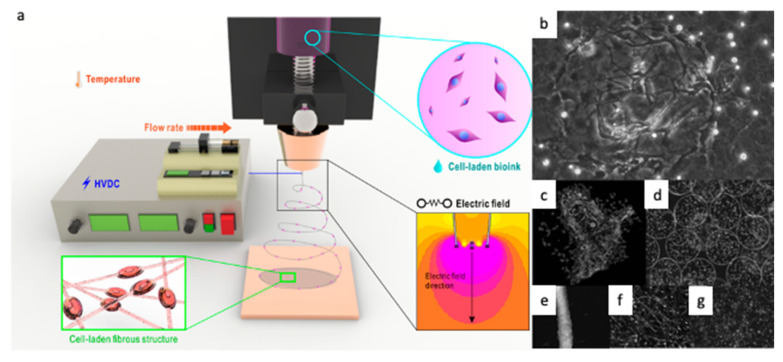
(**a**) Cell-electrospinning process with the basic components. Adapted from [79,91] with permission from MDPI. (**b**–**d**) Images of living structures fabricated using either BES or (**e**–**g**) CE. (**b**) Characteristic optical image illustrating a four cell culture system created in three dimensions (the image depicts cellular networks in three dimensions). (**c**) Confocal microscopy image of a three-dimensional culture prepared with the three major cell types of the myocardium (cardiac myocytes, endothelial cells, and fibroblasts). (**d**) Immobilized cells as composite living beads. (**e**) A vessel formed with cells embedded in the individual fibers. (**f**,**g**) The fiber configurations that could be altered from containing a single cell to a heterogeneous cell population. Adapted from [103] with permission from Wiley Materials.

Electrospinning has been a great advancement in the context of biomedical and tissue engineering applications. However, this technique presents some limitations, i.e., the use of cytotoxic solvents, and poor cell infiltration and distribution, since the cell seeding and incubation take place after the substrate processing. In order to overcome these limitations, a new methodology was developed called cell-electrospinning (CE), which differs from the conventional electrospinning on the use of living cells. CE consists of the application of the electrospinning process to a polymer solution combined with living cells, generating electrospun fibers with embedded cells. Figure 2a shows the schematic setup of the CE technique [90,91]. Jayasinghe et al. introduced this methodology for the first time in 2006. In this study, the authors were able to encapsulate living astrocytoma (1321N1) cells into polydimethylsiloxane electrospun fibers using a coaxial methodology. Cell viability, metabolic activity, and cell proliferation were examined and proven to be maintained for six days [90,92]. After this innovative study, the concept of CE was extended to different cell lines (stem cells, osteoblasts, cardiac myocytes, or neuroblastoma) and materials (polyvinyl alcohol (PVA), alginate, or Matrigel) [6,91,93,94,95,96,97]. Today, CE presents a breakthrough in polymer scaffolds processing, and offers remarkable opportunities in the area of biomedicine.

On the other hand, electrospray is a technique analogous to electrospinning that can be performed using the same device. The main difference between both techniques relies on the jet of the polymer generated after the high voltage application. In this case, the resulting jet suffers continuous break-ups, and the aerosolization of the solution takes place, resulting in the production of polymeric nanoparticles [98,99]. The properties and size of the particles will depend on the material and processing parameters. Particularly the viscosity of the polymer solution is a crucial parameter that can act as a switch between electrospinning and electrospraying [100]. Electrospray has been broadly used in different fields such as sensors, food processing, and biomedical applications due to its simplicity and ability to process different polymers. Even though it was developed before ES, today the use of electrospray is less common than electrospinning for the processing of polymer solutions.

Similarly to CE, the technique bio-electrospray (BES) was developed for the preparation of nano/microgels encapsulating living cells (images of living structures fabricated using BES are shown in Figure 2b,d). Jayasinghe et al. were also pioneers in using this technique in 2006 [92]. In this work, the group processed Jurkat cells obtaining deposited droplets in the range of tens of micrometers. BES does not influence cell viability, which has been verified on cell lines such as sperm or stem cells [101,102,103,104,105]. This methodology has been proven to be a useful tool for cell encapsulation, the controlled deposition of cells on planar surfaces, drug delivery, and immunotherapy [102,106].

The successful application of these techniques requires that the viability and bifunctionality of the encapsulated cells be not negatively affected during the process. Several conditions such as the solution’s viscosity, electric field applied, distance to the collector, or feed rate can influence the fiber size and shape, as well as the viability of the loaded cells. Therefore, controlling both material and processing parameters is essential to avoid stress damage of cells during the process, and therefore to provide high cell viability values of encapsulated cells. In this review, we extensively investigate the most important research studies carried out to fabricate cell-laden scaffolds using selected natural polymers and applying CE and BES technologies. It is expected that this review can serve as a reference tool and can give a better understanding of the methodologies for future research works.

### 3.1. Naturally Derived Polymers for CE and BES

Electrospinning and electrospraying techniques have shown great processability using both naturally and synthetically derived polymers. Synthetic polymers such as poly(dimethylsiloxane), polycaprolactone, or polylactic acid have been widely used for electrospinning and electrospray because of their versatility in the selection of the solvent that can provide adequate viscosity, conductivity, and surface tension of the polymer solution [91,106,107]. However, viability of encapsulated cells is highly affected by the use of the organic solvents commonly used, such as tetrahydrofuran, acetone, or chloroform [108]. Therefore, natural polymers such as alginate, collagen, and cellulose, compatible with non-toxic solvents, are frequently used for achieving biofabrication using living cells by cell-electrospinning and bio-electrospraying methodologies. Naturally derived polymers possess numerous advantages such as high cell affinity, low immunogenicity, or ECM biomimetic properties [109]. However, their low mechanical strength makes them challenging to process by electrospinning or electrospray [110]. These limitations can be overcome by blending natural polymers with synthetic polymers as well as modifying the processing methodology by employing a core-shell nozzle [109,111,112].

In this review, we will make an overview of the different biomaterials based on natural polymers and blends used for CE and BES techniques and the processing procedure used to obtain mechanically stable systems.

#### 3.1.1. Alginate

Alginate is a low-cost biodegradable polymer biocompatible with numerous cell lines, but it also presents a poor mechanical strength. Alginate was used by Xie et al. for entrapment of living cells by BES technology. In this study, the droplet formation was analyzed to optimize the production of monodisperse cell-laden microcapsules with controllable size. The electrospray procedure was performed in dripping mode, and an additional ring electrode was used to improve stabilization. This modified set up allowed the successful encapsulation of Hep G2 cells into calcium alginate microbeads with narrow size distribution and more controlled conditions compared to conventional electrospray [113].

Several works have prepared blends of alginate with other polymers to increase its electrospinnability. For example, Yeo et al. described a cell-electrospun system based on a blend of alginate, poly(ethylene oxide) (PEO), and lecithin to encapsulate MG63 osteoblast cells for their application in bone regeneration [6]. The concentration of the polymers as well as the electric field applied were optimized to ensure adequate cell viability as well as mechanically stable nanofiber mats. As a result, the highest cell viability for encapsulated osteoblasts (around 80%) was obtained with 2 × 10^5^ MG63 cells/mL, 2 wt% alginate, 2 wt% poly(ethylene oxide), and 0.7 wt% lecithin subjected to a 0.16 kV/mm electric field. Moreover, osteogenic differentiation of the cells was confirmed after 10 days of culture. Subsequently, hybrid scaffolds with high mechanical strength were prepared combining the cell-laden electrospun fibers and poly(e-caprolactone) microstructures prepared by 3D printing. It can be said that the cell-laden electrospun scaffolds enhanced the potential of 3D structures for bone regeneration, providing a high surface area and ECM-like structure.

Alginate/PEO blend was also used by Yeo et al. for encapsulating C2C12 myoblast cells by CE for skeletal muscle regeneration [114]. In this work, a high cell viability (around 90%) was obtained for encapsulated cells with an applied electric field of 0.075 kV/mm. It must be highlighted that alignment of the cell-laden fibers in the mat during the CE process allowed the achievement of highly aligned cells, which is proven to facilitate myogenic differentiation. Therefore, this study provides a new tool for achieving cell topographical cues by controlling fibers’ orientation, which can be very advantageous, especially for muscle regeneration.

**Figure 3:**
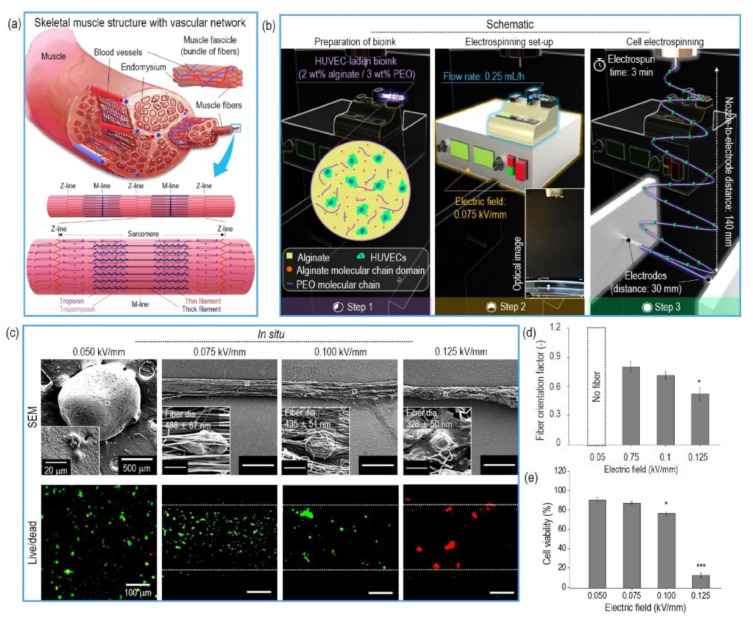
Schematics of (**a**) a native skeletal muscle structure with a vascular network and (**b**) the cell electrospinning process using human umbilical vein endothelial cells (HUVECs). (**c**) SEM and live/dead images of HUVECs-laden fibers fabricated using various electric fields. A quantitative analysis of (**d**) orientation factor of nanofibers and (**e**) cell viability where the analysis of variance (ANOVA) was used for the multiple comparisons and *p* * < 0.05, *p* ** < 0.01, and *p* *** < 0.001 indicate the statistical significance. Adapted from [115] with permission from Elsevier.

In order to go a step further in this direction, this group proposed a method to prepare a hierarchical platform with a topographical cue for co-culture of human umbilical vein endothelial cells (HUVECs) and C2C12 myoblasts cells [115]. An alginate/PEO blend was again used to develop aligned HUVECs-laden fibers by uniaxial CE, and cell viability at different electric fields was studied (schemes of native skeletal muscle structure and CE process, SEM and live/dead images, and quantitative analysis of orientation cell viability are presented in Figure 3a–e). Encapsulated HUVECs presented high cell viability (around 90%) at 10.5 kV of electrical field, homogeneous cell distribution, and efficient cell growth. The mat was combined with PCL/collagen struts prepared by 3D printing as a physical support. C2C12 cells were then seeded on the cell-laden fibers and co-cultured to facilitate myoblast regeneration. As a result, scaffolds containing HUVECs-laden electrospun fibers with a highly aligned topographical cue were able to enhance the myogenic-specific gene expressions.

#### 3.1.2. Gelatin

Gelatin is a collagen derivative with great biodegradability and biocompatibility, but low mechanical strength, which limits its fiber-forming ability [14]. Nosoudi et al. have demonstrated the successful production of cell-laden nanofibers using a gelatin/pullulan blend [116]. In this work, the electrospinnability of gelatin is enhanced by the presence of pullulan that increases the tensile strength of the blend. An 8 kV voltage and a concentration of 5 mg/mL gelatin/pullulan were used during the process, and adipose-derived stem cells (ADSCs) encapsulated within the fibers presented a 90% viability. This work offers a new area to be studied, since the use of gelatin for CE has been restricted until now by its mechanical properties.

#### 3.1.3. Fibrin

**Figure 4:**
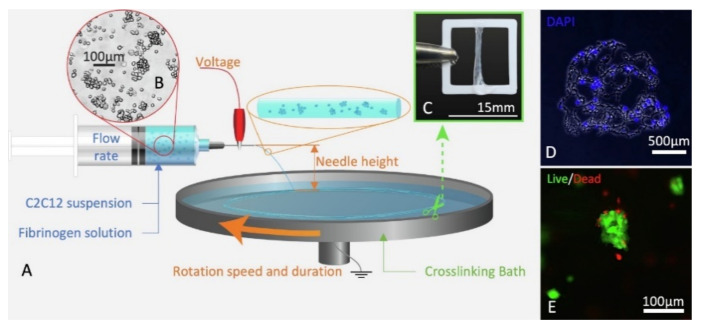
C2C12 can be electrospun into fibrin scaffolds. (**A**) Schematic of cell-laden wet-electrospinning setup identifying key parameters. (**B**) Bright field image of an aggregated cell suspension. (**C**) Cell-laden scaffold wrapped around ABS frame. (**D**) Cross-section of a cell-laden scaffold on Day 0 stained with DAPI (blue, nuclei). (**E**) High (20×) magnification of cell-laden microfiber bundles showing live (green) cells and dead (red) cells. For interpretation of the references to color in this figure caption, the reader is referred to the web version of this paper. Adapted from [17] with permission from Elsevier.

Fibrin matrix is formed by the polymerization of fibrinogen and thrombin in blood plasma. Due to good biocompatibility and fast biodegradability, it has been widely investigated for tissue engineering applications such as skin, cardiovascular, or musculoskeletal tissue regeneration. However, the mechanical properties of the fibrin matrix are very low. In a recent study by Guo et al., a fibrin matrix was used for cell encapsulation using CE technology [17]. C2C12s murine myoblasts were loaded as cellular aggregates (80–90 µm in diameter) into a fibrin/PEO polymer solution (schematic of CE process and cell suspension, cell-laden scaffold, and live/dead images are shown in Figure 4a–e). PEO was used to improve the mechanical properties of fibrin, as previously observed with alginate [6,114]. Electrospinning parameters were optimized to obtain homogeneous cells distribution inside the fibers and good proliferation after exposure to a 4.5 kV electric field and seven days of incubation. Moreover, myogenically induction provided elongated and multinucleated cells, demonstrating that encapsulated cells remained reactive to biological cues.

#### 3.1.4. Collagen

Collagen is one of the most abundant proteins in mammals and a main protein of the ECM. It is highly biocompatible and relatively non-immunogenic. Matrigel™ is derived from extracts of Engelbreth-Holm-Swarm mouse tumors and consists principally of collagen type IV, entactin, perlecan (heparan sulfate proteoglycan), and laminin.

CE technology was applied to a Matrigel-rich collagen biopolymer to encapsulate primary cardiomyocytes within fibers for the first time [117]. In this case, the applied voltage was 230 V, which resulted in cell viability values of around 80%, similar to the controls. Immunofluorescence staining exposed that the integrity of the encapsulated cells was maintained after the CE process. Combination of CE methodology with this biopolymer system allowed creating 3D cardiac patches that were demonstrated to enhance the cardiac tissue regeneration.

Matrigel with a high concentration of laminin was used in another work by Sampson et al. to encapsulate N2A mouse neuroblastoma cells [95]. In this study, CE technology was compared to aerodynamically assisted bio-threading (AABT). Samples prepared by CE presented cell viability values from 60% to 85% until three days of incubation. In vivo evaluation in mice was performed, demonstrating a good biocompatibility of the electrospun samples and the CE technique, compared to the control by AABT.

### 3.2. Future Trends

CE and BES are emerging biomedical techniques with great capabilities for living cells’ encapsulation into nano/microscale fibers. They allow the preparation of cell-laden scaffolds with high surface area and ECM-like structure using a simple methodology. Variation of both material’s and processing parameters can be controlled, which has demonstrated to have a direct effect on cell viability. Embedded cells have been proven to present good cell viability values and proliferation, and are responsive to cell cues. Moreover, alignment of the fibers can guide the cells to grow in the fiber direction. Therefore, these technologies have been demonstrated not to have a negative effect on cells for optimized processing conditions. However, some challenges still need to be addressed. Preliminary in vivo studies tested on animal models have demonstrated a good biocompatibility of CE, but more research studies are necessary to assess the efficacy and true applicability for tissue regeneration. Improvement of the mechanical properties of the mats and the cell density is still required. In addition, restrictions to developing 3D structures must be solved. These limitations can be overcome by combining these techniques with other biofabrication methodologies such as 3D printing. In this way, more complex scaffolds that are able to better simulate the complexity of native tissues can be achieved and the range of potential biomedical applications can be expanded.

## 4. 3D Printing

**Figure 5:**
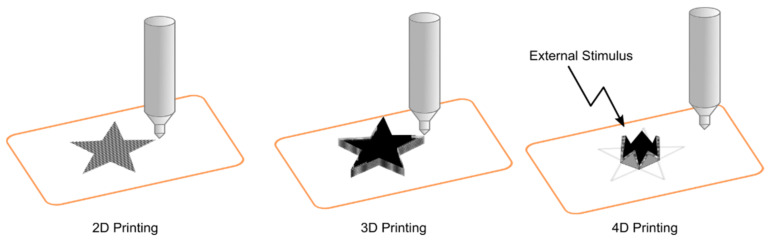
Differences between 2D, 3D, and 4D printing.

3D printing is a technology with the capacity to create objects by adding materials layer by layer using computer aided design (CAD) software [109,118]. This technology converts an object into sliced horizontal cross-sections that can be printed layer by layer to sort out the complete object in 3D (Figure 5). This technology allows the preparation of complex scaffolds for tissue engineering in a fast and low-cost way without the use of other expensive techniques [119]. One of the main advantages is the capacity to prepare low-volume scaffolds with the appropriate geometry to use in tissue engineering, allowing huge advances in implant materials and personalized scaffolds.

The development of new bioinks for 3D bioprinting has attracted attention in recent years. A bioink for biomedical applications can be defined as a formulation that can contain biologically active components and cells and is suitable for processing by an automated biofabrication technology [120]. The limitations of polymer-inspired bioink are toxicity, presence of toxic degradation products, and immune response between others. Recent studies have tried to develop new biocompatible bioinks and also polymer-free “bioink” consisting only of cells [121].

In recent years, 3D printing has been evolving into 4D printing. This technology is based on a shape transformation of the printed object in response to external stimulus, such as light, humidity, magnetic fields, enzymatic reactions, pH changes, or peptide detection [122,123] (Figure 5).

Therefore, functional 3D objects with the capacity to respond to biological conditions have been reported [123,124]. One of the main functions of 4D printing is the production of flexible-wearable biosensors with the capacity to detect small metabolites. In this sense, Nesaei et al. develop a bioink based on Prussian Blue and glucose oxidase enzyme solution to print two different microelectrodes that detect glucose in a concentration range between 100 and 1000 μM [125].

4D bioprinting also tries to include the use of cells to print living cellular structures with the capacity to evolve over time. The capacity to change the structure after receiving a stimulus could modify cell behavior and allow the formation of complex structures for tissue engineering [122]. For example, Kirillova et al. reported and advanced 4D bioprinting that allowed the fabrication of self-folding tubes based on hyaluronic acid and alginate. In this system, bone marrow stromal cells were encapsulated in a methacrylated alginate bioink and printed in different layers in combination with methacrylated hyaluronic acid layers. The system was crosslinked using a green light that is safe for the cells. Due to the difference in crosslinking degree between layers, the 3D bioprinted scaffolds have the capacity to fold forming tubes with the cells homogeneously distributed on the surface [126].

**Figure 6:**
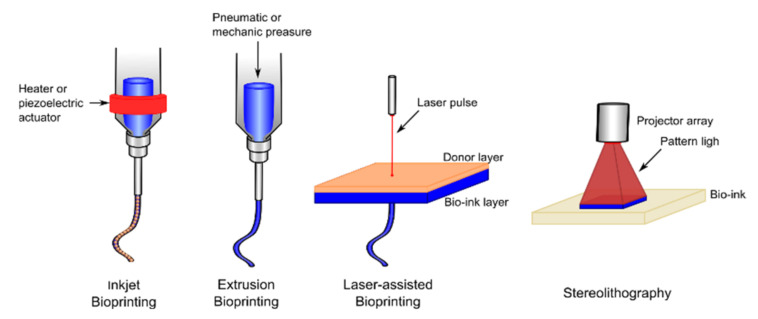
Schematic representation of bioprinting technologies.

The technology used for 3D printing is an important factor to determine the resolution capacity, velocity, and cell viability. The most important technologies used in the biomedical field are inkjet, extrusion, laser-assisted, and stereolithography bioprinting (Figure 6) [127].

Inkjet bioprinting is a droplet-based bioprinting system where the polymer solution in the chamber is extruded through a nozzle and droplets are generated on demand by the breaking of surface tension. The droplet can be generated using a thermal actuator, a piezoelectric actuator, or electrostatic forces. This technique only works with low viscosity liquids with low cell density [128].

Extrusion-based bioprinting is the most common and inexpensive technique. It is able to produce structures by staking multiple layers of a bioink by extrusion of a polymer solution through a micro-nozzle using continued pressure (pneumatic or mechanical) [129]. The properties of this technique are the capacity to deliver multiple cells and materials, i.e., high viscosity polymers with high cell densities, with a high cell viability.

Laser-induced forward transfer bioprinting consists of the deposition of a bioink layer that is in contact with a donor layer with the capacity to respond to a laser stimulation. During printing, a laser pulse is applied on the donor layer, and the bioink is propelled to the underneath substrate and immediately crosslinked [130]. This technique presents problems of cell viability due to the heating from the laser.

Finally, stereolithography uses light or laser to photolytically crosslink the bioinks layer by layer. This technique presents the highest resolution possible and high cell viability [128].

The reason for the increasing popularity of 3D bioprinting is the tremendous potential of the technique, which allows the production of tissues and other biological systems that mimic the in vivo tissue to repair. We are going to focus on the key points in 3D printing technology for biomedical applications: the development of new bioink and 3D printing for biomedical applications.

### 4.1. Recent Advances in Bioinks

Normally, the material used as bioink consists of natural polymers, cells, drugs, growth factors, and other materials that can be deposited in a controlled way. Bioinks should be non-toxic, easily printable, biocompatible, and biodegradable. We can define different families of natural polymers, as described in Table 2, that are commonly used for the preparation of bioinks.

The main problems of the commonly used bioinks are their low cell affinity and their limited mechanical properties. Recent advances in the development of bioinks are focused on the synthesis of derivatives that improve the mechanical properties and cell affinity and that provide signals to promote cell growth, adhesion, and differentiation.

#### 4.1.1. Alginate Based Bioinks

Alginate hydrogels have been extensively used as bioinks due to their good biocompatibility and similitude with the ECM. The easy way to use alginate as a bioink is by crosslinking with a solution of calcium chloride [144]. One of the main problems of alginates is their low printability and geometry accuracy due to their limited mechanical properties. To improve these properties, a variety of covalent crosslinking methods have been used. For example, Aldana et al. developed an alginate-based bioink with tunable mechanical properties using blends of alginate and gelatin methacrylamide (GelMA), obtaining a photopolymerizable biomaterial with different printability, accuracy, and mechanical and biological properties, depending on the ratio of alginate:GelMA [19].

A similar approximation was used by Soltan et al. The authors investigated the use of oxidized alginate (alginate dialdehyde, ADA) to obtain covalently crosslinked hydrogels with gelatin [145]. The mechanical properties of the hydrogel and therefore their printability and cell viability depend on the degree of oxidation and the ratio of ADA:gelatin. The authors printed different layers using two different cell types, human umbilical vein endothelial cells and rat Schwann cells, checking their viability over time.

In addition, the cell adhesion could be modified by the development of new blends. For example, the group of Boccaccini developed a hybrid hydrogel composed of alginate and keratin. This hydrogel promotes cell attachment, proliferation, spreading, and viability, being a good candidate for biomedical applications [146].

#### 4.1.2. Chitosan Based Bioinks

Chitosan has been widely employed in tissue engineering and biomedical applications due to its biocompatibility, biodegradability, and antimicrobial activity [147,148,149,150]. Normally, chitosan is crosslinked using genipin or glutaraldehyde by a chemical crosslinking mechanism [151]. Recent advances have been focused on the development of new crosslinking agents to improve the printability and accuracy of chitosan systems and, at the same time, add biological properties to the system to promote cell adhesion, migration, or differentiation. For example, the group of Prof. San Roman developed an ionic crosslinker based on phytic acid (G_1_Phy) for 3D printing [152]. This new crosslinker allowed the 3D printing of low concentrate chitosan/GelMA to obtain scaffolds with excellent mechanical and biological properties.

Another approximation to crosslink chitosan based bioinks for their use in laser-assisted bioprinting was developed by He et al. The authors provided a photocurable bioink based on the copolymerization of chitosan and acrylamide (AM) [153]. The capacity to use this bioink in laser-assisted bioprinting allows the preparation of complex 3D hydrogel scaffolds with high strength and good biocompatibility [154].

A different approach was presented by Puertas et al. in which the carboxymethyl derivative of chitosan was crosslinked with partially oxidized hyaluronic acid via Schiff base formation [155]. This study presented a novel bioprinting methodology based on a dual-syringe system with a static mixing tool that allowed the in situ crosslinking of the reactive hydrogel-based ink in the presence of living cells. This new approach allowed the use of low viscosity solutions while obtaining 3D printed scaffolds with good mechanical stability and proliferation of encapsulated cells.

#### 4.1.3. Other Natural Polymer Based Bioinks

There are a great variety of natural polymers that are being used as bioinks. In the previous topics, we showed different examples using GelMA in combination with alginate or chitosan. Other natural polymers such as hyaluronic acid, fibrinogen, agarose, collagen, or silk have been used as bioinks. Due to their bad mechanical properties or low cell affinity, these natural polymers need to be modified, crosslinked, or blended to obtain adequate properties for their use as scaffolds. For example, Skardal et al. developed a bioink based on GelMA and methacrylated hyaluronic acid. This bioink allows the direct incorporation of cells due to the good biological properties [156]. This group also developed another bioink based on the combination of fibrin and collagen with stem cells [157]. This bioink was used to print a full-thickness skin as a carrier of stem cells for the treatment of wound healing.

#### 4.1.4. Sacrificial Bioinks

Sacrificial bioinks are used to provide the necessary mechanical properties during the bioprinting step. After the scaffold is printed, the sacrificial bioink will be removed to create open spaces to allow cell adhesion and migration. Normally, water-soluble synthetic polymers are used as sacrificial bioinks due to their low adhesion to natural polymers.

**Figure 7:**
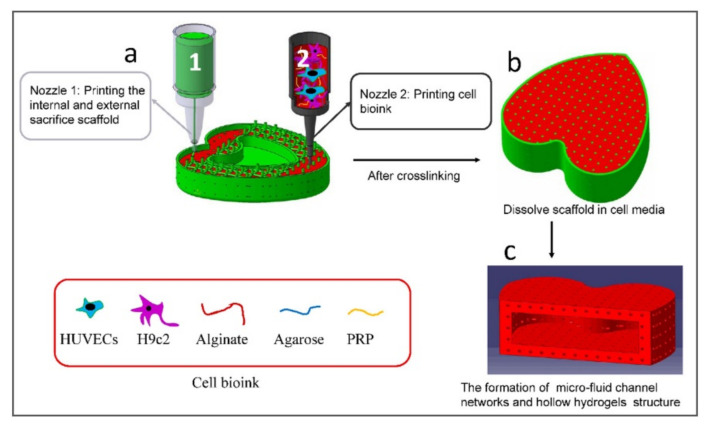
Flow diagram for the biofabrication of the large-size, hollow, and micro-fluid channel networks valentine-shaped heart. Reprinted from [158], with permission from Elsevier.

Synthetic polymers such as pluronic, PVA, or PEG are commonly used as sacrificial bioinks in natural polymers scaffolds like in the research of Zou et al. Here, the authors used PVA as a sacrificial bioink to prepare a porous scaffold of alginate agarose. First, a support scaffold of PVA was printed, and then the empty space was filled with an alginate/agarose/HUVECs bioink. Once the scaffold was finished, PVA was removed, with cell media forming a porous structure (Figure 7).

Important progress has been made in recent years in the use of sacrificial polymers. Jian et al. developed a bioprinting method using two different inks for meniscal reconstruction [154]. The system consists of two nozzles; one of them prints PCL by high-temperature melt deposition, forming the principal construct that provided the physical properties, and the second nozzle uses a mix of GelMa, ECM, and chondrocytes, and it is deposited in the free space between PCLs. Once the scaffold is finished, PCL only provides the necessary mechanical properties to the scaffold in the first days and is degraded, forming a microchannel in the scaffold that allows the transport of nutrients to the cells.

#### 4.1.5. Evolution of the Bioinks

The continuous research into new materials and processes promotes the fast evolution of the bioinks. In recent years, potential candidates have been emerging quickly. Decellularized ECM, self-assembling peptides, cellular aggregates, or nanobiocomposites have emerged in recent years [159,160,161,162].

From the point of view of the polymer field, the incorporation of nanomaterials into a bioink in order to improve the stiffness, shear-thinning, degradation, or stability is interesting. One of the most important nanocomposites is nanocellulose. Nanocellulose is derived principally from bacteria [163], and its use, combined with other bioinks, integrates the common properties of the cellulose: high stiffness, modulus, hydrophilicity, and thermal stability. For example, Han et al. studied the effect of nanocellulose on alginate/gelatin bioinks [164]. Their result showed that the incorporation of a small amount of nanocellulose improved the printability, stability, and fidelity of the structure, but high amounts of nanocellulose promoted a negative impact on the elongation and compression yield.

### 4.2. 3D Printing for Biomedical Applications

Current research on 3D printing in biomedical applications can be classified into the following areas: (i) printing of bioactive and biodegradable scaffolds and (ii) directly printing tissues and organs.

#### 4.2.1. Bioactive and Biodegradable Scaffolds

One of the main research fields in tissue engineering is the development of advanced scaffolds for tissue regeneration. In this case, the bioink will be a polymer system alone or a mixture incorporating cells, where the polymer systems play the role of the ECM. Compared with traditional scaffold-fabrication methods (salt-leaching, cryogels, or gas-foaming) that prepared simple-shapes supports with an inhomogeneity porosity, 3D printing can prepare complex structures, with the adequate shape to fill the defect and with an effective control of the porosity and microstructure. These systems require the presence of an interconnected porous network to allow cell growth and migration and flow transport of nutrients [120].

Bioactive scaffolds obtained by 3D printing can be divided into two families: scaffolds printed without cells (cell-free scaffolds) and scaffolds directly printed with cells (cell-loaded scaffolds).

*Cell-free scaffolds* are normally prepared from high water content polymer systems that present a high biocompatibility and a controlled biodegradation. The facility to prepared complex structures makes this kind of scaffold suitable for the reconstruction of complex tissues like osteochondral tissue. Osteochondral tissue is composed of different layers with different structures and compositions [25]. 3D printing is capable of producing scaffolds that simulate the structure of this tissue. For example, Gao et al. designed a multilayer system using GelMA with or without hydroxyapatite to obtain a scaffold that simulates the ECM in the osteochondral tissue [165]. The design of a proper bioink is a crucial point for the correct regeneration of the tissue. In this sense, Ma et al. developed a novel polymeric/ceramic bioink for customizing craniomaxillofacial bone reconstruction [166]. The bioink was based on a hyperelastic PEGylated urethane composited with microscale β-TCP. This bioink presented a high biocompatibility and osteoinductivity due to its biomimetic composition. In addition, it presented adequate mechanical properties for surgical manipulation and it had the capacity of osteoregeneration.

*Cell-loaded scaffolds* were developed due to the problems of seeding cells directly into 3D printed scaffolds (inhomogeneous distribution and inefficient cell adhesion). The main objective of this technique is to simulate the structure of the ECM in vitro to allow cell growth and differentiation. This technique allows the preparation of semi-functional tissues like in the case of the group of Prof. Jorcano. This group developed a functional skin by 3D bioprinting using extrusion bioprinting to deposit different layers composed of fibrin and fibroblast or keratinocytes to form the dermis and the epidermis layers [167]. With this technique, the authors obtained a functional skin that can be used for implantation in burned tissues.

#### 4.2.2. Directly Printing Tissue and Organs

**Figure 8:**
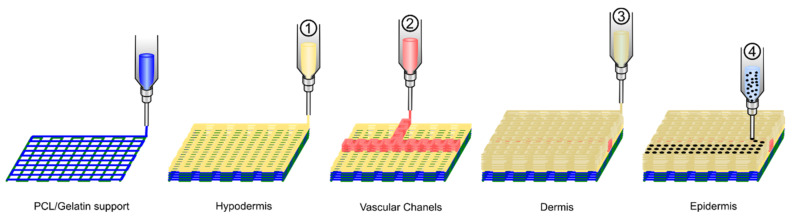
Schematic diagram exhibiting the 3D cell printing process for fabrication of a 3D full-thickness skin model.

The preparation of cell-loaded scaffolds does not assure the final functionality of the scaffold due to the dispersion of the cells and growth factors. For this reason, recent advances in 3D bioprinting are focused on the evolution of a direct-printing technology: tissue structures with physiological functions, containing seed cells, growth factors, and nutritional components [119]. One of the main goals is the pre-vascularization of the scaffolds, because the absence of a vasculature is one of the leading causes of failure for current 3D bioprinted scaffolds [168]. Recent advances have obtained functional vascularized tissues that showed better biointegration. Kim et al. developed a perfusable vascularized human skin formed by an epidermis, dermis, and hypodermis [169]. The system involves the preparation of a support of PCL and gelatin followed by the impression of the different skin layers: (1) The hypodermis layer was composed of fibrinogen and adipose-derived ECM with human adipocytes. (2) The vasculature was printed using a gelatin/glycerol/thrombin bioink with human umbilical vein endothelial cells. (3) The dermal layer was composed of fibrinogen, dermal-derived ECM, and human dermal fibroblast. (4) The final epidermis layer was composed of human keratinocytes. The result showed a fully functional skin with a microenvironment close to a real skin that can be used to test skin drugs or similar (Figure 8).

### 4.3. Future Perspectives in 3D Printing

Considerable advances have been achieved in the use of polymers for 3D bioprinting. However, this field is still in the early stages of development. One of the principal key factors is the development of a bioink adequate to our system. The bioink needs to have a good printability and geometry accuracy, adequate mechanical properties, and good biocompatibility and needs to be biodegradable. Many bioinks have already been formulated and used, but researchers continue to develop new compositions, looking to obtain better properties and new biofunctionalities. These characteristics allow the 3D bioprinting of fully functional tissues and organs.

Recently, 4D bioprinting technology has emerged as a powerful platform to obtain stimulus responsive bioprinting. This new methodology is in its first stages. Only a proof of concept with smart polymers has been developed. In the next years, this technology will evolve into more sophisticated systems and will be used in the advanced biomedical field.

## 5. Summary and Future Direction

In this review, we have entered into the relationship between natural polymers and new biofabrication techniques. The use of 3D printing, microfluidics, and electrospinning techniques has been widely investigated for the biofabrication of naturally-derived polymer scaffolds with encapsulated cells. Important challenges must be addressed for the successful biofabrication of these cell-laden natural scaffolds. On the one hand, the poor mechanical strength of natural polymers makes the processing and material manipulation difficult. In this sense, different modifications and blends have been investigated to improve the mechanical properties of the processed scaffold. On the other hand, the application of these techniques must ensure that the viability and functionality of the encapsulated cells are not negatively affected during the processing. Controlling both material parameters (e.g., solvent, viscosity, or polymer concentration) and processing parameters (e.g., pressure, voltage, or feed rate) is essential to avoid stress damage of cells during the fabrication, and therefore to provide high cell viability, metabolic activity, and proliferation of the encapsulated cells. Therefore, it is one of the main challenges that need to be addressed. In the last years, different modifications, blends, and adaptations of the biofabrication process have been investigated, but we are still in the first stages of the development of these technologies. There are indeed still many technological issues and limitations that need to be solved. One of the most important concerns is the clinical implementation of the cell-laden scaffolds fabricated using these techniques. Some preliminary in vivo studies in animal models have been performed. However, more research studies regarding immunological response and vascularization (which are essential for effective tissue implantation) are still necessary to assess the real applicability of the materials for clinical applications. Despite all these limitations and challenges, many efforts are being made to develop more complex techniques to simulate the complexity of native tissues and overcome the processing limitations, so we cannot exclude the possibility that in a few years biofabrication techniques will evolve and allow obtaining fully functional organs and tissues.

## Figures and Tables

**Table 1:** Summary of the studies exposed in this review regarding microfluidics generation of cell-laden microgels.

Polymer	Microfluidics Approach	Crosslinking Strategy	Microgel Size Range	Additives	Cell Type	Ref.
Alginate	Flow-focusing	Ionic crosslinking (Calcium-EDTA)	10–50 µm	No	MSCs	[48]
Alginate	Flow-focusing	Ionic crosslinking (calcium)	20–50 µm	poly-D-lysine	bMSCs	[49]
Alginate	Flow-focusing	Ionic crosslinking (Calcium-EDTA)	≈140 µm	PNiPAM	HepG2	[33]
Alginate	Centrifugal microfluidics	Ionic crosslinking (calcium)	Tunable (also fibers)	No	HepG2	[50]
Alginate	Double emulsion (w/o/w) flow focusing	Ionic crosslinking (calcium)	≤200 µm	Collagen	Hepatocytes and endothelial cells	[11]
Acrylamide hyaluronic acid	Flow-focusing	Enzymatic reaction and photopolymerization	≈80 µm	No	Human dermal fibroblasts	[8]
Furylamine and tyramine hyaluronic acid	T-junction	Enzymatic crosslinking, Diels-Alder click chemistry, or a combination	≈250 µm	MAL-PEG-MAL	ATDC-5 cells	[51]
N-carboxylic chitosan	Asymmetric cross-section	Schiff base reaction	≈200 µm	Oxidized dextran	NIH-3T3 fibroblasts	[52]
Chitosan Lactate	Flow-focusing	Ionic crosslinking (G_1_Phy and TPP)	100–130 µm	No	hMSCs	[45]
GelMA	Double flow-focusing	Photopolymerization	100–200 µm	No	macrophages	[20]
GelMA	Capillary	Photopolymerization	≈165 µm	no	bMSCs	[53]
GelMA	T-junction	Photopolymerization	300–1100 µm	PEGDA Poly(ethylene glycol)-fibrinogen	ECFCs breast cancer cells hiPSCs	[21]
GelNB	Capillary	Photopolymerization	300–600 µm	PEG-SH	bMSCs	[22]
Thiolated gelatin	T-junction	Thiol-Michael addition reaction	100–250 µm	Vinyl sulfonated hyaluronic acid	bMSCs	[16]
Dextran-tyramine	Flow-focusing	enzymatic crosslinking	120–200 µm	No	hMSCs	[54]
Dextran	Flow-focusing	Ionic crosslinking(calcium)	≈90 µm	PEG and Alginate	rat pancreatic islet	[55]
Methacrylated heparin	Flow-focusing	Michael addition	60–120 µm	PEG diacrylate monomers with 8-arm PEG-thiol	mESCs	[56]

**Table 2:** Most common natural polymers used for the preparation of bioinks.

	Compound	Advantages	Disadvantages	Bioprinting Technique	Ref.
Natural Polymers	Alginate	Low cytotoxicity, biodegradable, allow cell adhesion	Low mechanical properties	Extrusion	[19,130,131]
	Chitosan	Low cytotoxicity, biodegradable, antibacterial activity, allow cell adhesion	Low mechanical properties and depends on the origin and MW	Extrusion	[131,132]
	Gelatin	Lox cytotoxicity, improved cell adhesion, biodegradable	Poor mechanical properties and depends on the temperature. Low viscosity	Extrusion, Inkjet, Laser-assisted	[133,134,135]
	Hyaluronic acid	Similar to the ECM, biocompatible and biodegradable	Low mechanical strength and rapid degradation	Extrusion, Inkjet	[136,137,138]
	Collagen	Improved cell adhesion, good biocompatibility	Low mechanical strength and low viscosity	Extrusion, Inkjet, Laser-assisted	[139,140,141]
	Agarose	Good mechanical properties, biodegradable	Low cell adhesion	Extrusion	[142]
	Fibrin	Biocompatible, improved cell adhesion, non-cytotoxic	Low mechanical properties, rapid degradation	Extrusion, Inkjet	[143]

## Data Availability

Not Applicable.
